# On the Relations Which Exist between Human Tuberculosis and Bovine Tuberculosis

**Published:** 1899-01

**Authors:** Ed. Nocard

**Affiliations:** Professor at the Veterinary School of Alfort, France


### THE JOURNAL OF COMPARATIVE MEDICINE AND VETERINARY ARCHIVES.

Vol. XX. *JANUARY, 1899.* No. 1.

### ON THE RELATIONS WHICH EXIST BETWEEN HUMAN TUBERCULOSIS AND BOVINE TUBERCULOSIS.[1]

By Prof. Ed. Nocard,

PROFESSOR AT THE VETERINARY SCHOOL OF ALFORT, FRANCE.

*Translated from the "Annals of the Pasteur Institute," September*, 1898.

By Mazyck P. Ravenel, M.D.

*(Bacteriological Laboratory of the State Livestock Sanitary Board of Pennsylvania.)*

The identity of tuberculosis as seen in all kinds of mammifers is no longer questioned. There is still some discussion as to the relations existing between the bacilli found in tuberculosis of man and avian tuberculosis. Following Rivolta, Maffucci, Straus, and Gamaleia, many writers insist that these are two distinct species of microbes. Their arguments may be summed up as follows:

1. The appearances of the culture are different: Those of human tuberculosis are dry, scaly, hard to break up; those of avian tuberculosis are soft, thick, unctuous, folded, and are easily spread without much pressure; they still grow at 43° C., at which temperature the human bacillus ceases to grow.

2. As a rule, we cannot transmit the tuberculosis of mammifers to chickens, let the mode of inoculation be what it may.

On the other hand, certain mammifers are refractory to avian tuberculosis: The dog is one of these; the guinea-pig resists subcutaneous inoculation of the avian tuberculosis, and when it succumbs to intraperitoneal inoculation it shows lesions which are very different from those caused by the human bacillus.

Against these arguments we may object that, in spite of the difference in appearance, cultures from human and avian tuberculosis produce a tuberculosis which is identical; that sometimes we suc-

[1] Read before the Fourth Congress for the Study of Tuberculosis, Paris, July 30, 1898.

173529

ceed in producing tuberculosis in chickens by inoculating them with human tuberculosis, and that this tuberculosis is then inoculable in series (Ballinger, Koch, Nocard, Cadiot, Gilbert and Roger, Courmont and Dor); that there are some mammifers which are as susceptible to avian tuberculosis as to human tuberculosis, and after a few passages the lesions produced by one and the other are identical. Such is the case with the rabbit.

I have shown that it is the same with the horse. The horse, which is so resistant to experimental tuberculosis, becomes tuberculous quite frequently under natural conditions. The disease appears in it under two quite distinct clinical forms: In one form, much the most frequent, the lesions affect the organs of the abdominal cavity first, the intestines, mesenteric and sublumbar glands, the spleen, liver, kidneys, etc.; the lungs are affected in the last stage of the disease, and the pulmonary lesion is always of recent date. In the other form, on the contrary, the pulmonary lesion is the primary one; it is, moreover, of a very variable type, and only spreads to the abdominal organs very late in the disease. It is some time since I pointed out the differential symptoms of these two types of tuberculosis in the horse; quite recently I have been able to establish the etiology of these types which are so different clinically. While primary pulmonary tuberculosis is caused by a bacillus identical with that of human tuberculosis, it is a bacillus of the avian type, much modified by its passage through the organism of the horse, which provokes the abdominal type of tuberculosis in that animal.

The horse and the rabbit are not the only mammifers which are able to contract avian tuberculosis. A few years since I was able to study some tuberculous sputum inoculation of which, while rapidly fatal for the rabbit, rarely killed the guinea-pig, but the guinea-pigs which succumbed to the inoculation of this sputum showed at the autopsy lesions which closely approached those of avian tuberculosis. I was able to obtain cultures of this bacillus by sowing plentifully the pulp of the spleen of rabbits which had died after intravenous inoculation; these cultures were identical with those of avian tuberculosis. However, chickens inoculated with these cultures for the most part resisted, whatever the method of inoculation employed; but those which succumbed showed lesions like those of the natural disease. Here, again, the passage through the human organism had profoundly modified the virulence of the bacillus. Drüse, Pansini, and Johne have published analogous observations both in man and in cattle.

On the other hand, there are a certain number of well-attested facts on record where tuberculosis has appeared in barnyards several months after they have been put in charge of phthisical attendants. It seemed evident that the poultry became infected by eating the tuberculous sputa.

There are other analogous facts concerning chickens raised about abattoirs where they are allowed to feed on the condemned meats, of which tuberculous viscera always form the largest part. I am aware that Straus and Wurtz did not succeed in making chickens tuberculous by compelling them to ingest enormous quantities of the sputum of phthisical persons. For my part, I have not been more successful when obtaining the matter for ingestion from cows, pigs, or tuberculous horses. But can we be sure of obtaining experimentally all the conditions of natural contagion? Experiments are always carried out on only a small number of subjects, while in the cases cited the barnyards infected contained a considerable number, several hundred, for the most part. A single subject in a condition of least resistance may allow invasion by the bacilli from man or from cattle, and we can conceive how these bacilli, acclimated to the organism of the fowl, may in turn be transmitted more easily to the other inhabitants of the barnyards.

I have often discussed this question with my old friend, the much-lamented Professor Straus, but I was never able to shake his convictions. "I freely admit," he said to me, "the truth of the facts you bring forward; the rabbit, horse, cow, even man himself, can contract avian tuberculosis, like human tuberculosis. Does this prove that these tuberculoses are identical? When you have transformed the human bacillus into the avian bacillus I will avow myself convinced."

I believe that I have succeeded in doing this. I give the method:

We know how Metchnikoff, Roux, and Salimbeni have, in their fine work on the toxin of cholera, produced cultures *in vivo* in sacks of collodion. This ingenious method has quite recently enabled us to obtain pure cultures of the organism of contagious pleuro-pneumonia of the bovine species, which up to this time had baffled all research. It occurred to me to try this method with Koch's bacillus. This study is far from being completed; it has, however, already given some definite results on the subject-matter of my communication. I will recall briefly the principles of the process. Small sacks of collodion with very thin walls are prepared; after sterilization in the autoclave they are filled with bouillon previously sown with the germ or the virulent matter to be studied; they are closed securely, then introduced into the peritoneal cavity of a fresh animal, guinea-pig, rabbit, dog, sheep, cow, chicken, etc. One soon learns to execute all the manipulations without contamination, and not for an instant does the animal seem to suffer, either from the operation or from the presence of the sacks in the peritoneal cavity.

After a variable time, from a few days to several months, according to the character of the microbe studied, the animal is killed; the sack is found in some part of the peritoneal cavity, enveloped in a more or less thick layer of fibrin and cells of young fibrous tissue, from which we can easily enucleate it. When the experimental animal and the culture-liquid have been well chosen, surprising results are obtained, which, however, are easy to interpret. The wall of collodion affords an impregnable barrier both to microbes and the cells; the microbes cannot get out of the sack, but they are able to multiply there in perfect security, for the cells not being able to reach them, they are safe from phagocytic action. On the other hand, this wall, which is impermeable to microbes and cells, can be traversed by liquids and by substances in solution; it forms a perfect osmotic membrane, causing about it changes which profoundly modify the liquid first enclosed in it; substances elaborated by the microbes can diffuse themselves without, and when they are sufficiently active may lead to the death of the subject or to accidents of intoxication more or less grave, while not a single microbe has invaded the tissues; in any case it is a condition favorable to culture; microbial autointoxication is diminished if not suppressed. Finally, the products of the organism of the subject penetrate the sack, which may or may not be favorable to the growth of the microbe; the penetration of these products is always slow and gradual; little by little the microbe grows accustomed to the new medium, and when it is taken from the sack which has protected it from phagocytic action it is much better prepared to develop in the medium, at first hostile or refractory, which the incubator animal represents.

With these facts as a starting-point, I have attempted to obtain cultures of the bacillus of human tuberculosis in collodion sacks inserted into the peritoneum of the chicken. To succeed in this I have found it necessary: 1. To fill my sacks with a thick emulsion of a young culture, preferably obtained from glycerinated potato. 2. To open the sacks only after a minimum period of four months (the longer the stay of the sack in the peritoneum the more marked are the changes in the bacillus being incubated). 3. Finally, to operate on a sufficiently large number of chickens each time, rupture of the sack being more to be feared in proportion as the experiment is prolonged.

At the end of this work will be found a summary of my experiments, at least of those which I have been able to bring to a successful end before the assembling of this congress.

When the incubating animal is killed in order to regain the sack which has been inserted in it, the sack usually appears collapsed, more or less completely empty of liquid; it contains only a sort of slime, more or less thick, made up entirely of the bacilli of Koch. These bacilli are usually alive; when plentifully sown on glycerinated potato or agar they often give quite a feeble growth in isolated colonies, subcultures from which grow better and more quickly.

A curious thing is that when the collodion sack has remained for a long time in the peritoneal cavity of the chicken, the cultures obtained present all the characters of the avian type; they are soft, plentiful, unctuous, folded, easy to break up, and are easily spread on all media.

The characters of the original bacillus are thus profoundly modified by contact with the humors of the fowl. What has happened to its virulence? Inoculated under the skin the guinea-pig resists; sometimes a small abscess is formed at the point of inoculation, which opens anh heals spontaneously; sometimes, also, the related glands become hypertrophied and indurated, but the disease ends there. The pig remains well, it gains weight, and when killed no appreciable lesion is found.

If the inoculation is intraperitoneal, in more than half of the cases it will cause death. At the autopsy a fibrinous ascites is found; sometimes a crop of caseous granulations on the peritoneum; more often small bacillary purulent foci of the omentum; very frequently the spleen is enlarged in all dimensions, red, soft, friable, infiltrated by some large rounded nodules, white, caseous, or purulent. One never sees those well-marked degenerations which are characteristic of the Villemin type of tuberculosis in the guinea-pig.

The lung and the liver are generally unaffected. On the whole, the lesions resemble closely those produced by inoculation with avian tuberculosis.

The rabbit, inoculated in the aural vein, dies rapidly, in from six to ten weeks at most; it is very much emaciated; it shows at autopsy an intense miliary tuberculosis, most marked at times in the lungs, at times in the spleen. Let us inoculate a little of this splenic pulp into a second rabbit by the same method—it dies much more quickly, with lesions of tubercular septicæmia, with no apparent granulations, the pulp of the liver and spleen, and the marrow of the bones constituting, as it were, a pure culture of incredible richness of the bacillus of Koch.

These results are identical with those obtained by intravenous inoculation of cultures or of the products of avian tuberculosis. Chickens, on the contrary, resist inoculation of the contents of the sack, whatever the method of inoculation—ingestion, intraperitoneal, or intravenous.

It seems, then, that the modifications which the bacillus has undergone, and which are shown by the appearance of the cultures and the effects of inoculation on the rabbit and guinea-pig, are not sufficient to enable it to overcome the resistance of the chicken. Perhaps we may accomplish this by multiplying the passage in the collodion sacks so that the length of time during which the bacillus is in contact with the humors of the chicken, but protected from the phagocytes, will be doubled or trebled.

Let us again fill sacks of collodion with the young culture obtained from the first sacks, and put them into the peritoneal cavity of other chickens. After four, five, or six months we will find them, as before, half filled with bacillary mortar, a study of which will give much the same results as obtained before. In cultures we obtain growths identical with those of avian tuberculosis more certainly and more quickly. Inoculation of guinea-pigs and rabbits provokes lesions more closely resembling those of the avian type, but it remains without effect on chickens.

Let us repeat the experiment a third time: We will now accomplish our purpose. The bacillary sediment taken from the sacks after a stay of six to eight months in the peritoneal cavity of the chicken will be able to kill chickens (inoculated into the veins or peritoneum) with lesions identical with those of the natural disease.

In one of my experiments this transformation of the virulence of the bacillus was made quite naturally and much more quickly than in the period just given.

On January 11, 1897, a vigorous cock received into its peritoneal cavity a collodion sack filled with a young culture of bovine tuberculosis. (This culture, obtained from a case of tubercular mammatis, had all the characters of human tuberculosis; it killed rabbits and guinea-pigs with identical lesions; it had no appreciable effect on chickens).

For a long time the cock remained in good health, fat and vigorous; but all at once it began to waste away. This occurred toward the end of September. Its comb grew pale, flabby and soft, and fell to one side. Its condition grew worse, and the end seeming to be near, I killed him on October 28, 1897, nearly ten months after the insertion of the sack.

At the autopsy I found about the left testicle an irregular mass, about the size of a pigeon's egg, and sarcomatous in appearance, dotted with little caseous foci full of bacilli; the peritoneum was covered with tuberculous granulations, grayish or somewhat caseous at the central part; the liver and spleen were trebled in size and packed with like granulations. At the centre of the tumor I found the collodion sack, much broken at the point where it is joined to the glass. It seemed to me that the sack had been ruptured at a time when the bacilli, grown accustomed to live and grow in contact with the humors of the fowl, were able to resist the phagocytic action of its cells, and to cause the relatively recent tubercular lesions which the autopsy revealed.

No one could claim that this cock was tuberculous at the time of the insertion of the sack—that is to say, almost ten months before its death. Its long survival, the recent age of the lesions observed, which manifestly arose from the tumor formed around the ruptured sack, make such an opinion untenable. But what completely refutes it is the fact that two chickens which were inoculated with an emulsion of the splenic pulp of the tuberculous cock—one in the wing vein and the other in the peritoneal cavity—remained quite well, and when killed, on the 12th of May and 28th of July, 1898, showed no lesions whatever. If the material inoculated had been of avian origin these two fowls would have rapidly become tuberculous.

I trust that I have convinced you that it is possible to give to the bacillus of human tuberculosis the biological characters and the virulence which characterize the bacillus of avian tuberculosis. You will, then, conclude with me that these two bacilli, so different in appearance, are, however, only two varieties of the same species.

[NOTE.—In the original paper there follows an appendix, giving in detail the experiments, post-mortem notes, etc., which have been the basis of the above article. It has not been given here, since it is not of a character to interest the general reader, and the results obtained have been already fully given.]

